# Altruistic Vaccination: Insights from Two Focus Group Studies

**DOI:** 10.1007/s10728-022-00453-5

**Published:** 2022-12-01

**Authors:** Steven R. Kraaijeveld, Bob C. Mulder

**Affiliations:** 1 Philosophy Group, Wageningen University & Research, Wageningen, The Netherlands; 2 Strategic Communication Group, Wageningen University & Research, Wageningen, The Netherlands

**Keywords:** vaccination ethics, altruistic vaccination, empirical ethics, altruism, focus groups

## Abstract

Vaccination can protect vaccinated individuals and often also prevent them from spreading disease to other people. This opens up the possibility of getting vaccinated for the sake of others. In fact, altruistic vaccination has recently been conceptualized as a kind of vaccination that is undertaken primary for the benefit of others. In order to better understand the potential role of altruistic motives in people’s vaccination decisions, we conducted two focus group studies with a total of 37 participants. Study 1 included three focus groups on the subject of HPV vaccination for boys. Study 2 included three focus groups on the subject of pertussis and measles vaccination for childcare workers. We found substantial evidence of other-regarding motives across all focus groups, which suggests that altruistic motives could be an important factor when it comes to people’s vaccination decisions. We address the significance of these findings for vaccination policy surrounding HPV vaccination for boys and vaccination for childcare workers. We also extend the findings to normative work on vaccination for the sake of others more generally.

## Introduction

An important feature of preventive vaccination is that the health benefits can and often do extend beyond individuals who receive a particular vaccine (e.g., by preventing or reducing transmission to others or by contributing to herd immunity [1, 2]). Recent work on vaccination ethics has conceptualized different kinds of vaccination according to whether the underlying motives are more self- or other-directed; while vaccination may be guided by the goal of self-protection, it might also be undertaken primarily for the sake of others in what has been termed altruistic vaccination [3]. While altruistic vaccination may have health benefits for the person receiving the vaccine, the main impetus is to benefit (i.e., protect the health of) other people. Altruistic vaccination has been contrasted with indirect vaccination, which is when the decision for one person or group to get vaccinated for the sake of others is not taken by the vaccinee but by someone else, for instance when governments decide to implement vaccine mandates [3].

In order to gain a better understanding of altruistic vaccination and the dynamic between self-directed and other-directed vaccination motives, we conducted two focus group studies that centered on specific kinds of vaccination that appear to lend themselves particularly well to altruistic vaccination, because their potential benefits may be more substantial for people other than the individual vaccinees. More specifically, Study 1 examined the case of human papillomavirus (HPV) vaccination for boys, while Study 2 focused on the case of pertussis and measles vaccination for childcare workers (CCWs).^1^ These two subjects were selected because protecting the health of others appears to be particularly relevant in these areas, given that in both cases vaccination can considerably benefit others beyond individual vaccinees. Boys and men getting vaccinated against HPV can yield significant health gains for girls and women by helping to protect them against a number of HPV-associated cancers that are leading causes of death in women [4], while for pertussis and measles, vaccination by CCWs can help to prevent vulnerable children from contracting the respective diseases [5–7]. Vaccination for childcare employees against pertussis, for example, has been recommended to protect infants younger than 12 months old [8]. Aside from gaining insight into what people who may face choices regarding vaccination in these areas think about altruistic vaccination, we also sought to explore some of the normative implications of vaccination for the sake of others.

A research question was formulated for each respective study. First, what role do altruistic motives play for boys and parents of boys when it comes to accepting vaccination against HPV (for the sake of girls and women)? Second, how do altruistic motives factor into the acceptance of occupational vaccination of CCWs (for the sake of children)? Answers to these questions are important in order to better understand the motives behind vaccination that are relevant to the specific target groups, which can ultimately help to inform vaccination policy and feed into normative reflection. Should altruistic vaccination motives be found to be robust, for instance, then this would provide support for policies that cultivate such motives rather than for measures that potentially override them through more coercive measures, or ones that (merely) emphasize self-interest. Clarifying the role of altruistic motives will also contribute to theoretical work on the notion of altruistic vaccination. It can offer insight, for example, into whether people’s actual moral considerations and commitments regarding vaccination for the sake of others align with altruistic vaccination as a normative principle (i.e., that one ought to get vaccinated for the sake of others).

The paper is structured in the following way. First, we outline the methods employed for both studies. Second, we report the subjects and findings of Study 1, which examined HPV vaccination for boys. Second, we report the subjects and findings of Study 2, which addressed pertussis and measles vaccination for employees of daycare centers. Finally, we discuss the findings from the two studies within the context of vaccination policy and link them to more general normative-theoretical discussions surrounding vaccination for the sake of others.

## Methods

A focus group methodology was selected because focus groups are a productive means of stimulating discussion and of gathering a rich set of data on complex issues in bioethics [9]. Focus groups may lead to discoveries about the moral considerations for potential actions, and they can help us understand how and why participants make certain decisions; importantly, focus groups also tend to encourage people who might normally not speak up to contribute to a particular debate [10]. For each of the two studies presented in this article, we selected a total of three focus groups. This number is in line with study design guidelines for focus groups [11]. Having three focus groups per study was considered to be an effective means of organizing the discussions, as it allowed us to include a sufficient total number of participants for each study while having about 6 to 8 participants in each group, which is considered the optimum size for focus groups [12].

While all studies were intended to be conducted in person, the outbreak of a novel coronavirus disease (COVID-19) resulted in some parts of the studies having to be conducted online, including the session with young adults for Study 1 as well as all of the discussions for Study 2. Therefore, for Study 1, only the discussions with the two parent groups were conducted in person, while the discussion with the young adults was held via Skype chat. Using the chat function on Skype meant that all participants, including the moderator, exchanged text messages with each other in a single virtual chat room. We decided to use the chat function as it offered participants more time to respond and a higher degree of anonymity compared to speaking and listening through video. Furthermore, during the beginning of the first lockdown in the Netherlands, many people were not yet accustomed to online group meetings; we wanted to avoid the potential for technical issues and differences in ability to video call to negatively affect discussions. Audiotaping and transcription of the sessions were outsourced to external companies with respective areas of expertise. For an overview of the focus groups, see Appendices I and II.

All studies were conducted in March and April of 2020. The specific timeline for the studies is as follows. The three focus groups for Study 1 were conducted on March 2, March 3, and March 9. The first lockdown in the Netherlands in response to COVID-19 was implemented in the middle of march. The three focus groups for Study 2 were conducted on March 30, April 1, and April 6.

The focus groups were moderated by second author BM. A topic list was used to guide the discussion for each study. For Study 1, the list provided steps from the subject of altruism, vaccination in general, HPV, and specific considerations about whether or not to opt for HPV vaccination for children (see Appendix III). For Study 2, the list was the same, except the subject changed to pertussis and measles (rather than HPV) vaccination for the CCWs.

For each study, the discussion was initiated by asking the following very general question: “To what extent do you see people do things for each other?“ We decided to start the discussion by introducing the topic of altruism from a broad yet personal perspective, because we first wanted to explore the context in which people experience altruism (or a lack thereof) in their daily lives. This context of lived experiences shapes the factors that underlie attitudes towards altruism as well as other-regarding considerations relevant to vaccination. In particular, we sought to avoid introducing the polarization that characterizes much of the public debate surrounding vaccination early on in the discussion. By asking participants about quotidian experiences with regard to people doing things for each other (not yet explicitly naming ‘altruism’), we wanted to offer participants the chance to get to know each other and to acquaint themselves with how the discussion would proceed, in order to encourage the disclosure of personal opinions and experiences while also avoiding potentially controversial and confrontational topics right at the beginning of the discussion.

Participants were selected who were generally accepting of vaccination and who had previously participated in the national immunization program. People who are categorically against any kind of vaccination were not included for two reasons. First, in order to prevent discussions from being derailed by debates about whether one should get vaccinated *at all* (i.e., for any reason). We opted instead for a shared background among participants of at least a general acceptance of potential benefits of vaccination. Second, and relatedly, we wanted to examine altruistic vaccination specifically among people who might actually consider getting vaccinated for the benefit of others. We assumed that a person who is against *any* kind of vaccination on principle would not consider altruistic vaccination either. While it is certainly interesting and important to understand why people might be categorically against vaccination, this was not the focus of the present research.

In line with common practice [13], the coding scheme for the transcripts for both studies was partly developed *a-priori* (based on the literature) and partly developed *in-vivo* (based on the empirical input received from the focus groups). Transcripts were analyzed based on principles of thematic analysis [14], and using Atlas.ti software for coding. The analysis ran through several phases, in line with best practices [15]. First, both authors independently read the transcripts to immerse themselves in the data and for a general impression. Together with two graduate students, second author BM proceeded to code the transcripts, using the concepts from the topic list as *a priori* codes at the start of the coding process. Subsequent codes were developed *in-vivo* by assigning new and descriptive codes to quotations that were deemed relevant. In a next phase, BM and the graduate students compared coded transcripts to allow for emergence of different themes. Finally, first author SRK independently checked the identified themes against the transcripts. Consensus was reached by reviewing, integrating, and modifying the themes.

All research conducted for the purpose of this article was approved by the Social Sciences Ethics Committee of Wageningen University (number: 2020-6-Eerden), based on a review of the combined research protocols and materials. All participants gave signed informed consent before taking part in the focus groups and after being informed about its aims, the voluntary nature of participation, and the confidential treatment of all collected data. All transcripts were fully anonymized.

## Study 1: HPV Vaccination for Boys

### Subjects

All participants (n = 22) in the three focus groups conducted for Study 1 were recruited by a commercial agency specialized in recruitment and selection of research participants. From a database of over 25,000 participants, purposeful sampling was employed to organize three focus groups: one focus groups with parents from rural areas (n = 7); a second focus group with parents from the large city of Amsterdam (n = 9); and a third focus group (n = 6) with young adults aged 18 to 20 years, not specified as to residential area. The target groups were selected based on a recent study by the Dutch National Institute for Public Health and the Environment (RIVM) that recommends including male young adults as a target group for the Dutch national HPV immunization program [16]. Parents were also included, because parents have been found to play an important role in the vaccination decision of young adults [17]. The parent groups were further subdivided into parents from a rural area (‘rural parents’) and parents from an urban area (‘urban parents’), because previous research has indicated that there are meaningful differences in beliefs about HPV infection and vaccination between parents from urban as compared to rural areas [18, 19].

In terms of composition, we aimed for groups in which approximately a third of parents had at least one son, a third of parents had at least one daughter, and a third of parents had both a son and a daughter. The third focus group (‘young adults’) included a mix of male (n = 2) and female (n = 4) young adults aged 18 to 20 years. This group included both females who had already been vaccinated against HPV as well as those who had not. Even though the focus was on male young adults, female young adults were included as stakeholders in discussions surrounding HPV vaccination. As indicated in the Methods section, the discussions with the two parent groups were conducted in person, while the discussion with the young adults was held via Skype chat (for an overview of the focus groups, see Appendix I).

### Results

#### Altruism in General

The discussion was first directed by the moderator to altruistic behavior in general—to what it means to do something for someone else.^2^ This choice was made so as to approach the subject from a broad perspective and to avoid immediately shaping the discussion around vaccination.

Participants agreed that altruistic behavior can take various forms and can exists for a number of reasons. The most commonly offered example of altruism was voluntary work, or more specifically, volunteering to help others in need (e.g., family members, elderly people suffering from dementia, refugees, food banks). Historical changes in altruism were mentioned, but only by the rural parents, who suggested an increase in individualism over time as an explanation for decreased altruism within communities. Urbanization and increased general welfare were seen as a cause for the increasingly individualistic nature of contemporary (Dutch) society. Generational differences were mentioned by both rural and urban parents, emphasizing a decrease in altruism over time, but not by the young adults.

A consensus developed regarding the general complexity of striking a balance in life between benefitting others and benefitting oneself—between altruism and egoism. Participants considered it important to help others as much as possible, but they also agreed that one should not forget one’s own interests or “let others walk all over you” (male, rural parent).^3^ One young adult stated that helping others should not come at the cost of your own health and wellbeing. According to another young adult, “it is important to commit myself to others, but it is *me* before anything else.“ A rural parent, on the other hand, experienced trouble saying ‘no’ to people in need, which meant that she often put her own issues aside.

In all three focus groups, participants considered the status and nature of interpersonal relationships to be an important factor in altruistic behavior: knowing someone facilitated and made it more appropriate for them to be helpful, altruistic, and welcoming toward someone else. At the same time, they recognized that in some situations, like emergencies, whether or not you know someone personally is much less relevant.

Across the focus groups, three themes surrounding altruistic behavior were identified based on the discussions. The first comprises *self-regarding motives*, such as seeking to benefit in some way from the would-be altruistic act. Feeling satisfied with/about oneself was frequently mentioned as the most common way of ‘benefitting’ from an altruistic act. Social engagement and social inclusion were also commonly identified as ways of benefitting from helping others. Reciprocity or mutual benefit was also raised by several participants as a norm when it comes to helping others, but not everyone agreed. Interestingly, none of the participants mentioned negative emotions (such as feeling bad or guilty) or negative social consequences (such as punishment) as reasons to help someone.

The second theme involves *other-regarding* reasons, such as wanting to make others happy and to be useful to them, as well as empathic concern for other people. A general theme emerged of people wanting to be there for each other and to make others happy. Empathy was identified in the discussion as a potentially important factor underlying, and a means of generating, altruistic behavior. One rural parent explicitly offered empathy as a motive for altruism. A young adult mentioned that sometimes people help others under the supposition that their money is better spent on someone else who needs it more, which suggests empathy with the person in need.

The third theme encompasses *norm-based and duty-based* motives, such as helping someone else out of commitment to a particular group or organization. This theme was especially lively among the young adults, who mentioned helping other members of their student association as an example. Norms and values were generally considered important motives for altruistic behavior: most participants mentioned ‘norms and values’ either generically or with specific examples in relation to helping others. Doing things for someone else was considered ‘normal’ across all three focus groups; nevertheless, the source of the norms surrounding helping behavior was debated. Several participants claimed that helping others just feels like the right thing. Others insisted that helping others is part of one’s upbringing. One participant (female, urban parent) mentioned that her daughter does volunteer work, explaining that “to her [daughter] that is normal.“

#### Altruism and Vaccination

When it came to the subject of vaccination, the discussion centered more on the potential benefits to other people than to the vaccinee. Vaccination was seen by one parent as something that benefits not just individuals, but also society at large. Other parents responded that they had not expressly thought about vaccination in this way, but they agreed that is important for society that people get vaccinated in order to minimize the spread of diseases. Yet another parent related this idea to the outbreak of the novel coronavirus disease (COVID-19), which had made them more aware of the societal importance of vaccination. That vaccination by others can protect children who are undergoing chemotherapy, or who are otherwise unable to get vaccinated (e.g., due to allergies), was also brought up by participants. This resulted in a consensus among the parents that vaccination is important to protect vulnerable children, given that, as one parent pointed out, in every group there’s probably a vulnerable child. When it came specifically to boys getting vaccinated against HPV, one parent summarized the state of affairs as follows: getting vaccinated would mean avoiding that one contracts the virus that might result in ovarian cancer in girls and other cancers in boys. Both self-protective motives (for boys getting the vaccine) and altruistic motives (regarding girls and woman as potential sexual partners) were thus considered to be relevant by the parents.

Among young adults, this was also the case. Aside from self-protective motives, altruistic motives (i.e., protecting others) were clearly indicated in the willingness that was expressed to get vaccinated against HPV, considerations surrounding which mostly centered on males getting vaccinated for the sake of women. One young adult (male) told others, in a prelude to the discussion about HPV specifically, that he is receiving a meningococcal vaccine, which “prevents spreading of the disease, so that you increase the health of others.“ Another young adult pointed out that not every disease is contagious, so that benefitting others cannot always be a consideration. Nevertheless, the young adults agreed that when it comes to infectious diseases, vaccination means not just individual protection, but also protecting other people and society at large. According to one participant, “precisely because HPV is infectious, it seems to me that you can prevent it by collectively getting vaccinated,“ and “you naturally want to protect yourself, but also your sexual partner and the community.“

## Study 2: Pertussis Vaccination for Childcare Workers

### Subjects

All participants (n = 15) in the three focus groups conducted for Study 2 were recruited by a commercial agency specialized in recruitment and selection of research participants. The target groups consisted of CCWs who were actively working in childcare centers within the Netherlands at the time of the study. From a database of over 25,000 participants, three focus groups were selected. The first group (n = 4) included participants from across the Netherlands; the second focus group (n = 6) included participants from the city of Amsterdam; and the third focus group (n = 5) included participants from the region of Amersfoort. The participants’ age ranged from 24 years old to 61 years old (*µ* = 38,9 years). The participants’ work experience ranged from 1 year to 27 years (*µ* = 11,5 years). Of the fifteen participants, three were men. All of the discussions took place online, via Skype chat. For an overview of the participants, see Appendix II.

As indicated in the Methods section, all of the discussions took place online via Skype chat. For an overview of the participants, see Appendix II.

### Results

#### Altruism in General

As in Study 1, the discussion was first led to the subject of altruistic behavior generally, before moving to specific considerations in relation to vaccination. With regard to the question of why people help or do things for others, CCWs agreed that it is good to help people when one is in a position to do so. People help each other because it makes them feel good; helping others gives one a sense of accomplishment and feelings of happiness. Some self-regarding motives were thus identified for other-regarding behaviors; helping others can sometimes result in a benefit to the self by generating positive emotions and by making one feel better about oneself. The CCWs, however, concluded that for them the good feeling is not the most important outcome—it is only a bonus. The consensus was that it is normal and self-evident to sometimes engage in behavior for the sake of others.

Three other themes surrounding altruistic behavior were identified. First, the theme of what may be called *altruism out of love*, which entails doing something for another person because you love that person and care about them. Second, there was the theme of *reciprocal altruism*, or engaging in altruistic behavior in hopes of being the recipient of other people’s altruistic behavior in the future (also referred to as ‘karma’ in the discussion). Third, there was the theme of *altruism as a personality*, which involves embracing altruism as part of who one is as a person—explicitly adopting it as part of one’s personality. Participants in focus group three in particular mentioned that they thought that CCWs are more caring than most people, and possess the innate quality of altruism, which is why they work in childcare and “from the heart,“ according to one participant.

#### Vaccination Beliefs

The CCWs general views of vaccination were unequivocally positive. Participants first highlighted the protective effects of vaccination for them individually; when the discussion moved to occupational vaccination, the focus shifted to the beneficial effects for the children with whom they worked. One participant summed it up as follows: “I think that that vaccination is important so that children are protected [from diseases] and can grow up in a safe environment.“ One participant agreed that it is important to vaccinate young children against infectious diseases, suggesting the metaphor of “building as strong a wall as possible” around vulnerable children with children who have gotten vaccinated.

The positive views of vaccination remained throughout the focus group session, although the CCWs became more critical of vaccination as the discussion moved toward risk perception and the explicit importance of vaccination in order to protect others. At this point, it became clear that the CCWs felt that protecting others through vaccination is part of one’s professional role; they considered it to be part of their responsibility in caring for the children.

#### Altruism in Vaccination

Altruism was not found to be an independent theme for CCWs when it comes to accepting vaccination for the sake of others. Altruism was instead considered to be part of the responsibility of being a CCW, which includes protecting others through vaccination. In contrast to altruism in general, altruistic vaccination was not linked to generating a good feeling; it was primarily identified by participants with the idea of protecting others and connected to the notion of responsibility. Altruism and altruistic behavior were often taken for granted and seen as the unquestioned norm in their line of work: the CCWs found it self-evident that people would take the responsibility to protect the children with whom they work. As one CCW put it, “I take zero risks with my own children, so I also don’t [take risks] with someone else’s child.“

This sense of responsibility toward the children at their childcare center was shared by all participants. Protecting the children constituted a major reason for them to get vaccinated. This sense of responsibility was rooted in their protective feelings toward the children, as well as the expectation that one should do what is best for them. Participants agreed that CCWs are held responsible for the children, and also want to feel responsible: “This is also the reason that we work with children. We care about them, and we do not want to make them ill.“

#### Responsibility and/or Autonomy

One point of ambivalence developed in the discussion about vaccination and (moral) responsibility. Occupational vaccination was considered to be “something you just do, because you want to protect the children you work with. You wouldn’t want something to happen to them.“ Vaccines were held to “exist for a reason,“ according to one participant. And yet, the value of autonomy when it comes to making the choice of accepting or refusing vaccination was regarded very highly by the CCWs. The responsibility of protecting others and/or maintaining their own autonomy was identified as a source of moral tension: while accepting that vaccination is more or less self-evidently an important good linked to their responsibility as CCWs (i.e., a good for the children under their care), they nevertheless also greatly valued having autonomy over this decision and thus also having the option to potentially refuse vaccination.

The importance of shared responsibility regarding vaccination was also a talking point. Participants often mentioned that vaccination was not solely their responsibility. In their opinion, one is only able to protect others when everybody takes their societal responsibilities seriously and accepts vaccination. This extends to the responsibility of parents having their children get vaccinated, as well as that of the government to promote and facilitate vaccination.

Autonomy over the choice to get vaccinated also plays a role in shared responsibility: when people have individual autonomy, but fail to act according to their societal responsibilities by refusing vaccination, the CCWs consider it more justified if the government limits autonomy by, for instance, introducing a mandatory vaccination program. The CCWs would find mandatory vaccination more acceptable to the extent that it would (1) protect themselves and others, and (2) likewise apply to people in professions similar to theirs. These results suggest, then, that for CCWs altruistic vaccination is not so much an individual as a wider, societal choice. Given that success of getting vaccinated for the benefit of others depends more on larger groups of people than any given individual, CCWs consider it a shared responsibility of all of those involved to get vaccinated in order to protect the health and well-being of children.

## Discussion

### Central Findings Concerning Altruistic Vaccination

For both the target groups of HPV vaccination for boys (Study 1) and pertussis and measles vaccination for CCWs (Study 2), altruistic motives were generally embraced by participants. Across the two studies, participants were accepting of the idea of getting vaccinated in order to protect the health of others. The findings from the focus group discussions thus support the notion of altruistic vaccination [3], and are in line with previous research that has found altruistic motives to play a role in vaccination decisions and acceptance among other populations (e.g., healthcare workers and occupational physicians) [20, 21].

One of the central findings is that altruistic motives, especially in the form of a willingness to protect the health of others, were an important factor for people to potentially accept vaccination within each respective area. While there were some participants for whom altruism or protecting others were not major considerations in deciding whether or not to get vaccinated, people largely agreed that the potentially positive effects on the health of other people, on the whole, constitutes an important good and is a morally relevant reason to consider getting vaccinated.

While participants sometimes spontaneously brought up the positive effects of vaccination for others, as well as the importance of these positive effects for vaccination decisions, this more frequently occurred across the two studies only after the moderator pointed out these potential positive effects. Two main conclusions can be drawn from this. First, that altruistic motives (e.g., to protect the health of others) are on the whole considered morally relevant to vaccination decisions by the target groups, at least within the specific areas of potential vaccination considered in this paper. Second, that this idea—i.e., that other-regarding effects of vaccination are morally significant—is not always spontaneously arrived at by people when they consider vaccination. Nevertheless, people were generally responsive to normative arguments for altruistic vaccination when these were suggested to them by the moderator. This suggests that, when it comes to vaccination policy, it is imperative to explicitly address the health benefits to other people of the vaccines in question, above and beyond the positive effects on the health of the individuals (or more directly the self) who would be receiving the vaccine. As the findings show, people tend to be receptive to such altruistic considerations, even if they had not previously recognized them. This demonstrates the importance of tailoring information to what we know about the substantive motives of a given target group, and it suggests that an approach that overtly engages with these motives may be more effective than approaches that attempt to bypass more rational deliberation (e.g., nudging), although more research is needed in this area.

The intrinsic motivation to protect the health of others through vaccination was present among the target groups that we examined, which means that mobilizing altruistic motives may be vital when it comes to promoting vaccination for the sake of others in these areas. Although we did not explicitly study altruistic attitudes toward COVID-19 vaccination, future research should investigate these in order to examine whether a similar picture emerges. Some research to date does suggest that altruistic motives matter for COVID-19 vaccination [22–25]. Of course, the idea of getting vaccinated for the sake of others (i.e., to reduce the chances that one would transmit a disease post-vaccination), especially if it is to be used to motivate people to get vaccinated, has to be sensitive to the actual affordances of different vaccines. Different vaccines will, for instance, be associated with various levels of effectiveness. This has been one of the issues surrounding COVID-19 vaccines, which, according to the most recent evidence, have only a modest and temporary effect on reducing transmission [26, 27]. This makes arguments and communication strategies that rely on other-directed effects more difficult and less convincing than for vaccines that have more robust effects on reducing transmission. The specific effects of particular vaccines are important to take into consideration when it comes to the ethics of vaccination policy, especially when coercive measures are involved [28]. Indirect vaccination strategies^3^ must also take into account the potential effects of vaccines on reducing transmission and the possibility of achieving herd immunity. To give but one example, while some have argued that children should get vaccinated against influenza for the sake of elderly people [29], in the case of COVID-19, it has been argued that such a strategy is currently not ethically justified [30, 31]. One related issue, which also arose in the context of HPV vaccination, is that, while influenza vaccines are not very effective for elderly people who are particularly vulnerable to influenza, COVID-19 vaccines do seem to be effective for elderly people and even for people who are immunocompromised [32, 33]. When vulnerable target populations can effectively protect themselves, this generally weakens the reason that others have to get vaccinated for their sake—although people may still wish to do so, as demonstrated by the discussion surrounding HPV vaccination for boys . Even if girls and women can get vaccinated against HPV themselves, boys and men may still have a reason to get vaccinated even beyond the individual benefits.

### Ambivalence and (Moral) Conflicts

In each study, there was some ambivalence between different moral considerations when it came to getting vaccinated for the sake of others. The specific nature of this ambivalence—and the degree to which it might be considered to be a moral conflict—differed substantively between the two studies.

#### Egoism vs. Altruism

In Study 1, the main source of ambivalence concerning HPV vaccination for boys was between what may be called egoistic and altruistic motives—between getting vaccinated to protect one’s own health and/or getting vaccinated for the good of others (specifically, potential sexual partners). The relevant self-regarding and other-regarding motives were clearly distinguished in the discussions for the target groups. However, the discussions never quite reached the point of uncovering a moral tension, given that participants—parents and young adults alike—tended to see the moral value *both* of boys/men protecting themselves against HPV through vaccination *and* of males thereby also ultimately protecting girls/women. Therefore, one cannot strongly delineate an either/or situation or moral conflict here, because individual interests were not seen as being strongly juxtaposed against the interests of others. In the end, self-interest and the interests of others were more or less aligned for the target groups in the case of HPV vaccination.

#### Freedom vs. Coercion

The situation was different in Study 2. The main area of tension when it came to getting vaccinated against pertussis and measles for CCWs was between the moral value of retaining one’s autonomous choice in vaccination decisions and/or protecting others (i.e., the children under one’s care) by getting vaccinated. For the CCWs, a moral conflict emerged, which can be formulated as follows. On the one hand, there was consensus regarding the importance of protecting children by getting vaccinated, meaning that there were strong motives to protect others in this way. On the other hand, the CCWs greatly valued the freedom to make their own vaccination choices , and they were troubled by potential mandates that might limit their decisional freedom. One aspect of this conflict that did not wholly surface in the discussion, but which may be important as a partial explanation, is that responsibility for the health of the children under their care—and especially *taking* responsibility in this area—was crucial for the CCWs. Participants indicated in the discussions that they prided themselves in taking responsibility for the children’s well-being. Yet, one can only *take* responsibility for something when there is a genuine choice to be made—when there is a real opportunity to exercise one’s moral agency. Should vaccination decisions become subject to coercion, for instance through vaccine mandates, then this would limit the CCWs (perceived) ability to take responsibility for protecting the health of the children under their care.

With regard to vaccination policy, this finding about conflicting moral values among CCWs provides a compelling case for leaving CCWs free to make their own vaccination decisions. To the extent that the values of CCWs ought to be respected, vaccination policy should avoid overriding their choice to get vaccinated for the sake of the children. The results of the discussions suggest that it would generally be better to encourage pertussis and measles vaccination and to make the vaccines readily available and accessible without, however, going so far as mandates. Given that CCWs are already inclined to want to protect children in their care by means of vaccination, but that they hesitate when faced with potential coercion, such mandates may ultimately backfire by generating resistance and reactance. Mandates may undermine the extant altruistic motives of CCWs to care for the children in their charge (cf. [34]). Similarly, mandates can also undermine trust [35].

Nevertheless, some room for mandates was left open by the CCWs. They would consider mandatory vaccination to be more acceptable if it (1) protected them and others, and (2) also applied and extended to people in professions similar to theirs. Perhaps a mandate in some form may ultimately be acceptable to CCWs, as long as policies are in place to ensure that (at least) their own acceptability criteria for mandatory vaccination are met. This is interesting to consider in relation to different potential approaches to vaccination for the sake of others that turn on varying degrees of decision freedom and coercion [3]. An altruistic approach would leave CCWs free to get vaccinated against measles and pertussis (or not) in order to protect children, and would encourage policies that refrain from enforcing the decision in some way. Based on the findings and the foregoing discussion, this ultimately appears to be the approach best suited to the CCWs themselves.

In Study 1, the discussion did not explicitly turn to mandatory vaccination. The young adults as well as the parents recognized the moral significance of protecting one’s sexual partner against contracting HPV through vaccination and also of thereby minimizing the spread of HPV among the community more generally. The motives revealed in this study may also be understood within the context of altruistic vaccination, even if self-protective motives were likewise widely considered. The current study does not lead us to a conclusion regarding mandatory HPV vaccination for boys in order to protect girls and women, but it does suggest that stressing the benefits to others is potentially important for boys and their parents to opt for vaccination against HPV. Personal responsibility in any case weighs more heavily against mandates for some vaccines, given that it is easier for people to control the transmission of some infectious diseases than others [36]. Since HPV is only transmitted through intimate skin-to‐skin contact, “a sexually active teen or adult who receives regular screenings for sexually transmitted infections can radically reduce her chances of infecting others,“ meaning that the ethical justification of coercion is significantly weaker than for more readily transmitted diseases [36]. Moreover, whereas vaccination for CCWs would be undertaken primarily for the sake of vulnerable children who cannot (yet) be vaccinated, a similar dynamic does not hold in the case of HPV vaccination, where girls/women can get vaccinated as well as boys/men. In the end, girls and women can also protect themselves against HPV by getting vaccinated. The health benefits of HPV vaccination are thus less exclusively obtained by one of the parties getting vaccinated. This weakens the case for more coercive measures, given that there is still the option for girls and women to get vaccinated against HPV and thus protect themselves against HPV-related cancers, even if greater public health benefits may ultimately be achieved when all parties (i.e., girls and boys) get vaccinated.

### Social Norms and Moralization

An interesting aspect of the discussion among CCWs was that they would consider coercive mandates to be more acceptable should they also be extended to other people in similar professions. There has been recent debate about the moralization of vaccination decisions, which may at times be morally inappropriate and which may have pervasive negative consequences [37]. It could be that the reluctance of CCWs to be singled out for vaccine mandates at least partly reflects their perception of moralized social norms for their particular occupation. Even though they were not specifically asked about moralization, the fact that CCWs so clearly perceived vaccination to be the dominant social-moral norm suggests that vaccination of childcare workers may be moralized. To what extent moralization plays an active role, and to what degree it stems from people within their own field or is imposed on them from society at large is an important question for future research. In any case, it speaks to a sense of fairness when it comes to vaccination policy: CCWs did not believe that mandates would be fair at least unless others, in similar professions, would also be subjected to them.

### Communication Considerations

Effective public communication is vital to the success of vaccination programs [38]. Our findings suggest that tapping into altruistic motives by stressing the health benefits of vaccination to others beyond the self should be part of this communication infrastructure. Our results build on previous findings that employing altruistic frames and informing people about the social benefits of vaccination (like community protection) can increase vaccination intentions [39]. If the goal is to increase vaccine uptake, then only providing information about vaccines and vaccine-preventable disease may not be sufficient [40]. Given that participants did not always realize the benefits of vaccination to others, yet were receptive to such benefits when they were pointed out to them, communication about vaccination should stress other-regarding considerations—especially in the case of vaccines for which the benefits to others are expected to be particularly substantial. As previously discussed, in the case of COVID-19 this strategy may not be as effective, given the more limited vaccine effectiveness in terms of preventing infection and transmission of the virus.

### Limitations

There are at least three potential limitations to the studies presented in this article. First, the participants may not have felt entirely free to express socially undesirable opinions (e.g., more selfish attitudes) within their respective discussion groups. It should be noted that this is a limitation of focus group studies more generally [41]. However, the discussions with young adults about HPV vaccination were anonymously conducted, which mitigates concerns about social appearance at least for those findings. The freedom with which participants expressed themselves anonymously did not appear to differ substantively from in-person discussions, which further attenuates this concern. Finally, given that there was at least one instance where a participant explicitly expressed what might be considered a socially undesirable attitude (i.e., qualifying their concern for others with the statement that is “*me* before anything else”), this suggests that people did not structurally feel prohibited from expressing more ‘selfish’ attitudes.

Second, all of the studies were conducted in the Netherlands, which means that our findings cannot be taken to represent people’s attitudes in other settings. Cross-cultural comparison was, however, beyond our present scope. One interesting aspect to point out is that previous research has shown that participants from countries with a collectivistic background (e.g., South Korea) were more likely to express prosocial vaccination attitudes compared to those from a more individualistic cultural background (e.g., the U.S.) [42]. That prosocial vaccination attitudes were found to be robust in a sample of people from a relatively individualistic country like the Netherlands [43] suggests that collectivism may not be necessary for the emergence of such attitudes, although comparative research is necessary to examine this idea more rigorously.

Finally, people who are categorically opposed to vaccination were excluded from the current research, which may be seen as a limitation with regard to the representativeness of our findings. However, since we were interested in the idea of altruistic vaccination, we reasoned that people who would not get vaccinated for *any* reason would also, by extension, not get vaccinated for the sake of other people. Perhaps the discussions in the focus groups would have progressed differently had people who are strongly opposed to vaccination been included; but the dynamic that might have ensued would tell us more about how people opposed to vaccination might influence those who are generally accepting of it, than it would about the views of those who might at least in principle be willing to get vaccinated for the benefit of others.

## Conclusion

We found evidence across two focus group studies for the presence of altruistic vaccination motives. In Study 1, protecting the health of girls and women was generally considered to be an important motive for boys to get vaccinated against HPV. In Study 2, protecting the health of vulnerable children was a widely shared motive for childcare workers to get vaccinated against pertussis and measles. Altruistic motives for vaccination thus played an important role for the respective target groups. These findings underscore the significance of the idea of altruistic vaccination and suggest that vaccination policies, at least for HPV vaccination for boys and pertussis and measles vaccination for childcare workers, should highlight the potential health benefits to others. Given that people are sensitive to the moral importance and the moral good of those benefits—even if they had not previously realized it—public health strategies to increase vaccination for the sake of others should tap into people’s altruistic motives . This approach is preferable to more coercive measures, since it respects the autonomy and values of the different target groups examined in this paper and stands to reinforce rather than undermine extant altruistic motives.

## Appendix I: Overview of Participants in Study 1

**Table:**

Focus Group 1 (In person; Parents; Rural Living Area)			
	*Gender*	*Children (Age in Years)*	
Y1	Female	Sons (6 & 10)	
Y1	Female	Son (12)	
Y3	Female	Son (15)	
Y4	Female	Sons (11, 14, & 16)	
Y5	Male	Daughters (16 & 20)	
Y6	Male	Daughter (21) & Sons (11 & 18)	
Y7	Female	Daughter (12) & Son (10)	
Focus Group 2 (In Person; Parents; Urban Living Area)			
	*Gender*	*Children (Age in Years)*	
Y1	Female	Daughter (1) & Son (15)	
Y2	Female	Daughters (3 & 8) & Sons (13 & 14)	
Y3	Female	Daughter (22) & Sons (12 & 21)	
Y4	Female	Daughter (15)	
Y5	Female	Son (10)	
Y6	Female	Daughter (10)	
Y7	Male	Daughter (10) & Son (14)	
Y8	Male	Daughters (11 & 11)	
Y9	Male	Daughter (8) & Sons (12 & 15)	
Focus Group 3 (Young Adults; Online)			
	*Gender*	*HPV Vaccination Status*	*Living Area*
Y1	Female	Vaccinated	Urban
Y2	Female	Vaccinated	Urban
Y3	Male	N/A	Urban
Y4	Male	N/A	Rural
Y5	Female	Not vaccinated	Rural
Y6	Female	Not vaccinated	Urban

## Appendix II: Overview of Participants in Study 2

**Table:**

Focus Group 1			
*Gender*	*Age*	*Years of Employment*	*Household Size*
Female	31	9	2
Female	27	7	1
Female	61	11	4
Female	55	25	3
Focus Group 2			
*Gender*	*Age*	*Years of Employment*	*Household Size*
Female	50	5	4
Male	32	2	5
Female	35	8	3
Female	43	22	5
Male	39	10	4
Female	59	10	4
Focus Group 3			
*Gender*	*Age*	*Years of Employment*	*Household Size*
Male	33	8	2
Female	24	4	4
Female	43	19	3
Female	25	1	4
Female	26	2.5	1

## Appendix III: Topic List Focus Groups

Setup

- Introduce researchers
- Discuss goal of the research
- Create a safe and open environment

Introduction

- Who are you? First names, age, where people are from
- Do you have a son, daughter or both? How old are they? (Study 1)^4^

Altruism

- Do you see people do things for each other?
- Why do people choose to do things for one other?
- What are the lessons you want to teach your child(ren) with regard to doing things for other people (and egoism)? (Study 1)

Vaccination

- What is your attitude toward vaccination?
- What is the role of society in vaccination?
- What is your attitude toward vaccination of your child(ren)? (Study 1)
- What is your opinion about vaccination being an altruistic act?

Specific Questions about HPV (Study 1)/Measles and Pertussis (Study 2)

- What is HPV (measles/pertussis)?
- What is your attitude toward vaccination (for male young adults/measles and pertussis)?
- What are reasons to let your child get vaccinated against HPV? (Study 1)
- What are reasons to not let your child get vaccinated against HPV? (Study 1)
- What information about HPV (measles/pertussis) vaccination are you missing?
- Where would you like to find this information?
- What is the government’s role or position in the HPV (measles/pertussis) vaccination debate?
- What is your attitude toward the risk perception of HPV (measles/pertussis)?
- Is there a higher chance you will have your child get vaccinated against HPV (or will get vaccinated against measles/pertussis yourself) if there is a relatively high chance of them (or children in daycare) getting or transmitting the virus?
- Is there a higher chance you will have your child get vaccinated against HPV (or will get vaccinated against measles/pertussis yourself) if there is a relatively high chance of them (or children in daycare) getting or transmitting the virus?

(Ethical) Considerations

- How important are the benefits to others for considerations toward the HPV (measles/pertussis) vaccine?
- What is your attitude towards the HPV vaccine, now that you know that your child(ren) will also be protected if someone else will get vaccinated against HPV? (Study 1)
- What is your opinion about parents’ decisions not to let sons get vaccinated against HPV? (Study 1)
- What is your opinion about letting children decide (or letting CCWs decide) to get vaccinated on their own?

## Footnotes

1 It should be noted that the two studies were part of a larger project, which also included topics that are not reported in this paper.

2 The discussions were moderated by second author BM.

3 The discussions were conducted in Dutch; all quotations throughout the article were translated into English by first author SRK.
